# Influence of Hypoxic Preservation Temperature on Endothelial Cells and Kidney Integrity

**DOI:** 10.1155/2019/8572138

**Published:** 2019-06-04

**Authors:** Sébastien Giraud, Clara Steichen, Pierre Couturier, Solenne Tillet, Vanessa Mallet, Rémi Coudroy, Jean-Michel Goujon, Patrick Hannaert, Thierry Hauet

**Affiliations:** ^1^INSERM U1082, Poitiers 86000, France; ^2^CHU Poitiers, Service de Biochimie, Poitiers 86000, France; ^3^Université de Poitiers, Faculté de Médecine et de Pharmacie, Poitiers 86000, France; ^4^IBiSA Plateforme “Plate-Forme Modélisation Préclinique, Innovation Chirurgicale et Technologique (MOPICT)”, Domaine Expérimental du Magneraud, Surgères 17700, France; ^5^CHU Poitiers, Service de Réanimation Médicale, Poitiers 86000, France; ^6^CHU Poitiers, Service d'Anatomopathologie, Poitiers 86000, France; ^7^University Hospital Federation Tours Poitiers Limoges “Survival Optimization in Organ Transplantation”, CHU Poitiers, 86000, France

## Abstract

Ischemia-reperfusion (IR) injury is unavoidable during organ transplantation and impacts graft quality. New paradigms are emerging including preservation at higher temperature than “hypothermia” or “cold”: although 4°C remains largely used for kidney preservation, recent studies challenged this choice. We and others hypothesized that a higher preservation temperature, closer to physiological regimen, could improve organ quality. For this purpose, we used an* in vitro* model of endothelial cells exposed to hypoxia-reoxygenation sequence (mimicking IR) and an* ex vivo* ischemic pig kidneys static storage model.* In vitro*, 19°C, 27°C, and 32°C provided protection against injuries versus 4°C, by reducing cell death, mitochondrial dysfunction, leukocyte adhesion, and inflammation. However,* ex vivo*, the benefits of 19°C or 32°C were limited, showing similar levels of tissue preservation damage.* Ex vivo* 4°C-preserved kidneys displayed a trend towards reduced damage, including apoptosis. Macrophage infiltration, tubulitis, and necrosis were increased in the 19°C and 32°C versus 4°C preserved kidneys. Thus, despite a trend for an advantage of subnormothermia as preservation temperature, our* in vitro* and* ex vivo* models bring different insights in terms of preservation temperature effect. This study suggests that temperature optimization for kidney preservation will require thorough investigation, combining the use of complementary relevant models and the design of elaborated preservation solution and new technologies.

## 1. Introduction

A significant fraction of kidney graft dysfunctions observed after transplantation is due to unavoidable ischemia-reperfusion (IR) injuries (IRI), which impact short- and long-term transplantation outcomes [1]. Indeed, despite extracorporeal “cold” preservation period which is used worldwide to overcome this issue, graft injuries are frequently observed and caused by pathophysiological mechanisms related to ischemia and severe hypothermia. Furthermore, because of the severe organ shortage, donor demography has changed and additional sources of organs are grafts recovered from extended criteria donors (ECD) and donation after circulatory death (DCD) [2, 3]. However, these organs are particularly exposed and sensitive to IRI and therefore more prone to develop dysfunctions. Although transplantation of marginal grafts demonstrates promising outcomes, increased rates of primary nonfunction, delayed graft function, and reduced graft survival have been reported [3]. Cold ischemic injury, caused by static cold storage, is a significant risk factor for poor outcome. In this context, optimization of organ preservation protocols is a pivotal goal.

Currently, kidney preservation procedure is based on hypothermia: grafts are flushed and conditioned at 4°C in a preservation solution, such as University of Wisconsin solution (UW), Custodiol, Solution de Conservation des Organes et des Tissus (SCOT-15), and Institut Georges Lopez-1 (IGL-1). However, this preservation procedure is being questioned by scientific community and numerous articles highlighted that hypothermia indeed worsens ischemic injuries through (i) reduction of ATP synthesis and metabolic activity [4], (ii) reduced Na-K-ATPase activity, which induces osmotic perturbation [5, 6], (iii) mitochondrial perturbations, (iv) decreased cell survival [7, 8], and (v) endothelial activation [9, 10].

There are emerging concepts of alternative storage temperatures, which could limit IRI and recent studies advocate the use of normothermia, mild hypothermia, and subnormothermia [2, 11, 12]. Preclinical findings have ignited clinical organ preservation research that investigates dynamic preservation, its various modes (continuous, preimplantation), and temperatures (hypo-, sub-, or normothermic).

Applied to mouse livers, normothermic (37°C) ischemia reduced sinusoidal perfusion, increased leukocyte adhesion to the endothelium and altered tissue integrity. However, temperatures of 26°C, 15°C, and 4°C reduced these injuries equally [13]. Compared to normothermia, mild hypothermia (32°C) reduced infarct size on rabbit hearts undergoing IR [14]. Moderate hypothermia (30°C) decreased myocardial energy utilization during ischemia and subsequently promoted expression of proteins involved in cell survival, compared to 34°C [15]. In a mouse model of partial liver ischemia of 90 min performed at various temperature (4°C, 15°C, 26°C, 32°C, and 37°C), oxidative stress was reduced at 15°C to 32°C [16]. Rat liver perfusion temperature of 21°C showed an improvement of hepatic function recover, compared to 4°C or 12°C [17]. In addition, mild hypothermia (34 to 35°C) applied to organ donors significantly reduced the rate of delayed graft function [18].

Regarding normothermic preservation, different techniques are described. One of the proposed methods used an acellular normothermic perfusion system [19]. An alternative technique, developed by Nicholson and Hosgood, used an adapted pediatric system with a packed red cell based solution [20]. Novel strategies are emerging using machine perfusion at subnormothermia and normothermia levels [2]. Other approaches must be also taken into consideration such as hypothermic or normothermic abdominal regional perfusion in high-risk donors with extended warm ischemia times [21] or controlled rewarming after hypothermia [22, 23]. These are new principles and practices, which bear promising features and which ought to find a place in renal preservation. Collectively, these reports suggest that an intermediate temperature might be more efficient to protect organs against IRI, particularly from marginal donors.

We designed the present study to assess the effects of different temperature levels during preservation. We focused on targets demonstrated to be critical in the pathophysiology of cold ischemic and reperfusion injuries: endothelium, mitochondrial dysfunction, cell death, and activation of innate immunity and inflammation [24, 25]. Because our policy is to minimize the use of* in vivo* models, we designed alternative models in order to establish a* corpus* of consistent data, allowing clarifying specifications for organ preservation at different levels of temperature. First, we used an i*n vitro* model of IR (hypoxia-reoxygenation) applied to porcine primary endothelial cells, the endothelium being the first* in situ* histological component to be exposed during IRI. A second set of experiments was designed to evaluate the effect of preservation temperature* per se* in a preclinical,* ex vivo* porcine kidney preservation model. Indeed, the anatomic and physiologic similarities between pigs and humans, especially for the kidney, make this model a valuable and relevant translational model [26].

## 2. Materials and Methods

### 2.1. Porcine Endothelial Cells

Endothelial cells were prepared from cortex porcine renal tissue of a 3-month-old large white pig (MOPICT plateforme, INRA, Surgères). The remaining tissue was digested using 1 mg/mL Collagenase IA (Sigma Aldrich, France) + 200 *μ*g/mL DNase I (Roche Diagnostics, France). Nonspecific binding was blocked with porcine IgG (Interchim, France). Cell suspension was incubated with anti-CD31 antibody conjugated to dynabeads (Fischer-Bioblock, France) and then isolated by immunomagnetic separation (Dynal, Fischer-Bioblock, France).

### 2.2. In Vitro Hypoxia-Reoxygenation Model of Ischemia-Reperfusion

Porcine renal endothelial cells were cultured on Dulbecco's minimum essential medium (DMEM), supplemented with 10% fetal bovine serum, 1% L-Glutamin, and 1% pyruvate (all from Fischer-Bioblock) (completed DMEM), at 37°C with 5% CO_2_. Cells were used at passages 4 to 5. For preservation, cellular “ischemia” was achieved by incubating cells in a hypoxic atmosphere: 0% O_2_, 5% CO_2_, and 95% N2 (Bactal 2 gaz; Air Liquide, France) for 24 hours at 4°C, 19°C, 27°C, 32°C, or 37°C in UW solution. “Reperfusion” was performed either by replacing UW for 24 hours in completed DMEM or for 2 hours in anticoagulated porcine blood at 37°C in 20% O_2_, 5% CO_2_, and 75% N2. Control cells (CTL) were incubated in completed DMEM at 37°C in 20% O_2_, 5% CO_2_, and 75% N_2_.

### 2.3. Oxygen Measurement

Confluent endothelial cells cultured in flask were incubated in a anoxic atmosphere: 0% O_2_, 5% CO_2_, and 95% N_2_ (Bactal 2 gaz; Air Liquide, France) for 24 hours at 4°C, 19°C, 27°C, 32°C, or 37°C in UW solution. Partial pressure of oxygen was measured in real time with oxygen probes connected to the OXY-4 fiber optic oxygen transmitter (PreSens, Precision Sensing GmbH, Regensburg, Germany). At the different hypoxic incubation temperatures, measurements of oxygen were carried out in the atmosphere chamber and simultaneous in the atmosphere culture flask and in the UW preservation medium during the hypoxia period.

### 2.4. Lactate Dehydrogenase Release Quantification

At specific time points, supernatant and cells were collected to quantify lactate dehydrogenase (LDH) levels using an analyzer (Modular analytics P; Roche Diagnostics, Meylan, France).

### 2.5. Succinate Dehydrogenase Activity Assay by XTT

Following the different culture conditions, cells were incubated with XTT labeling mixture (XTT kit; Roche) following manufacturer's instructions and reaction was quantified by spectrophotometer (Victor3; Perkin-Elmer, France).

### 2.6. ATP Assay

We used ATP Bioluminescent Assay Kit (Sigma) following manufacturer's instructions. Briefly, ATP is consumed, and light is emitted when firefly luciferase catalyzes the oxidation of D-luciferin.

### 2.7. Reverse Transcription and Real-Time Reverse-Transcriptase Polymerase Chain Reaction

Total RNA isolation was performed with NucleoSpin® RNA-XS kit (Macherey Nagel, France). Genomic DNA was removed using DNA-free kit (Applied Biosystems) and first-strand reverse transcription (Applied Biosystems) was performed to obtained cDNA. Specifics porcine primers were designed using OligoPerfect™ (Invitrogen), sequences detailed in Table S1. Amplification was conducted on an automated analyzer ABI PRISM 7300 (PE Applied Biosystems, France). PCR amplification mix was 2 *μ*L cDNA at 5 ng/*μ*L + 2 *μ*L forward and reverse primers (500 nmol/L final concentration) + 10*μ*L SYBR green (SYBR® green PCR Master Mix, Applied Biosystems) + 6 *μ*L ultrapure H_2_O. PCR reaction was performed following manufacturer's instructions (hold: 10 min at 95°C, 40 cycles: 15 sec at 95°C, 1 min at 60°C, and finally melt). Finally, mRNA expression level in the sample was obtained with the comparative Ct method (and expressed relative to control, normalizing the target gene to an internal standard gene, L19 (RPL19)): 2-∆∆Ct, where ∆∆Ct = (Ct,T - Ct,R) _x_ - (Ct,T - Ct,R) _cb_, with Ct,T the threshold cycle of the target gene and Ct,R the threshold cycle of the standard gene and where *x* refers the experiment point and* cb* to the calibrator point.

### 2.8. Leukocyte Adhesion

After 24 h of hypoxia at various temperatures, ischemic porcine endothelial cells were reoxygenated 2 h with anticoagulated (Li-heparin) porcine blood at 37°C. Two hours after incubation, cell monolayer was washed 3 times in phosphate-buffered saline to eliminate blood red cells and no-adherent leukocytes, and refringent adherent leukocytes on monolayer endothelial cells were counted in culture wells, under microscopic evaluation.

### 2.9. Electron Microscopy

Cells samples were processed for transmission electron microscopy, as previously described [27]. Cells pellets were fixed in 3% glutaraldehyde, washed, and postfixed in 1% osmium tetroxide. Then pellets were dehydrated in acetone and embedded in araldite. Ultrathin sections were cut and stained with uranyl acetate and lead citrate and were examined under an electron microscope (JEOL 1010, Tokyo, Japan). The degree of lesions was determined by using a semiquantitative graded score: 0 = no alteration, 1 = lesions between 1-25%, 2 = lesions between 26-50%, 3 = lesions between 51-75%, and 4 = lesions >76% (percentages of lesions by field).

### 2.10. Ex Vivo Experiments

The surgical and experimental protocols were performed in accordance with the ARRIVE guidelines, with EU Directive 2010/63/EU, and with institutional Poitou-Charentes Ethical Comity of animal experimentation (protocol number: CE2012-4). We used 3 months old large white pigs weighting 40 ± 4 kg (IBiSA plateforme, INRA, Surgères, France). After analgesia/anesthesia, surgical procedures were performed to remove the right kidney, which was flushed with UW solution (Bristol-Myers-Squibb, France) at various selected temperatures (4°C, 19°C or 32°C) and preserved during 24 hours under static conditions at the selected temperature.

### 2.11. Histological and Immunohistological Study

Biopsy samples from corticomedullary kidney sections were fixed with 4% formalin and paraffin-embedded. Renal histological injuries were quantified using Periodic Acid Schiff (PAS) coloration. The degree of histological lesions was determined in the cortex by using a semiquantitative graded scale. Brush border loss, tubular dilatation, and endoluminal detachment were assessed using a semiquantitative 6-points scale: 0 = no alteration, 0.5 = lesions <5%, 1 = 6-10%, 2 = 11-25%, 3 = 26-50%, 4 = 51-75%, and 5 = >76% of lesions.

The number of infiltrates in the renal tissue was quantified using PAS staining. Quantitative determination of tubulitis was adapted from Banff classification: 0 = no mononuclear inflammatory cell in tubules; 1 = 1 to 4 mononuclear cells* per* tubular cross-section; 2 = 5–10 mononuclear cells* per* tubular cross-section, and 3 = >10 mononuclear cells* per* tubular cross-section.

For immunohistochemistry study of macrophage infiltration in the renal tissue, slides were deparaffinized, saturated with a specific buffer (Dako protein block X0909, from Agilent, Les Ulis, France), and incubated with primary antibody for 2 hours in a humid atmosphere (MAC-387 antibody ab80084, from Abcam, Cambridge, United-Kingdom, used at 1/100 in Dako antibody diluent S2022, from Agilent, Les Ulis, France). Secondary antibody was incubated at room temperature for 30 minutes (HRP anti-rabbit K4002 ready to use, from Agilent, Les Ulis, France).

For immunofluorescence study to quantify the number of cleaved caspase-3 positive cells in the renal tissue, slides were deparaffinized, saturated with a specific buffer (Dako protein block X0909, from Agilent, Les Ulis, France), and incubated with primary antibody for 2 hours in a humid atmosphere (Caspase 3 cleaved antibody MAB835, from R&D Systems, Lille, France, used at 1/200 in Dako antibody diluent S2022, from Agilent, Les Ulis, France). Secondary antibody was incubated for 1 hour at room temperature (Anti-rabbit 488, A11034, from Thermofisher, Illkirch, France, used at 1/1000).

Quantification of the number of apoptotic cells in the renal tissue was performed using the DeadEnd™ Fluorometric TUNEL System kit (staining of DNA fragmentation), according to the manufacturer's instructions (Promega, Charbonnières-les-Bains, France).

All evaluations were performed and scored under blinded conditions by an anatomopathologist.

### 2.12. Statistical Analysis

Results were expressed as percentage of level obtained in “IR simulated” cells over control living cells. Results are expressed as mean ± SEM. For multigroup comparisons we used the Kruskal-Wallis test and Dunn's posttest; differences were considered significant if p < 0.05.

## 3. Results

### 3.1. Oxygen Measurement

**Figure 1:**
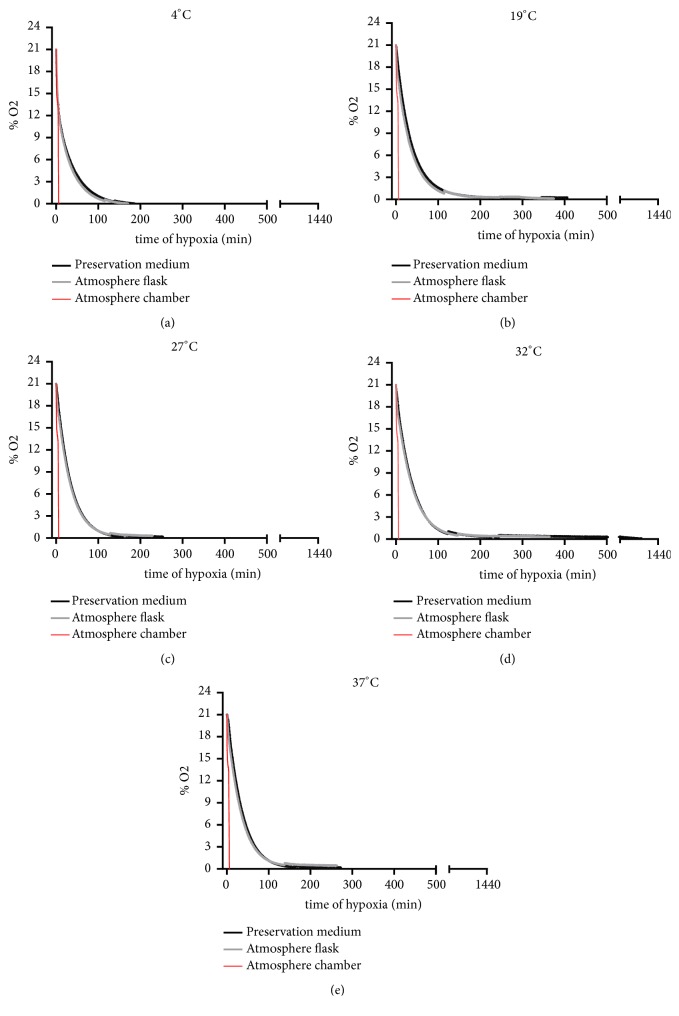
Effects of various preservation temperatures on oxygen pressure level during induction of hypoxia by Bactal 2: partial percentage of oxygen were measured in real time in the chamber atmosphere, in the cell culture flask atmosphere, and in the UW preservation medium during the hypoxia period, at 4°C (a), 19°C (b), 27°C (c), 32°C (d), and 37°C (e). Measured values of oxygen (atmospheric%) are expressed as the mean value of n = 3 determinations per group.

First, oxygen was reduced to 0% in the chamber atmosphere within 6.5 min after induction of hypoxia by the use of Bactal 2 gas. Independently of the incubation temperature, oxygen pressure level as measured both in the flask atmosphere and in the cell culture medium were reduced simultaneously and below 1% after 130 min of incubation with Bactal 2 (Figure 1; n = 3* per* group).

### 3.2. Necrosis at the End of Hypoxic Preservation

**Figure 2:**
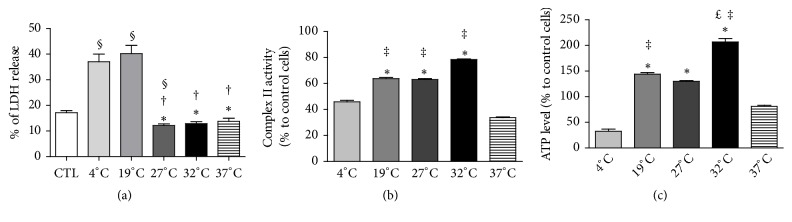
Effects of various preservation temperatures on endothelial cells viability and on the mitochondria. (a) LDH release, measured at the end of the hypoxic preservation, is expressed as LDH release in supernatant/ intracellular LDH (U/L). Results are expressed in mean ± SEM (n = 8 per group). CTL: control cells not subjected to any manipulation. The integrity and activity levels of mitochondrial respiratory chain complexes were measured after normothermic reoxygenation: (b) complex II (n = 16 per group); (c) ATP production by Complex V (n = 16 per group). Results are expressed as mean ± SEM (% of control cells). Statistical significance (p<0.05) were calculated using nonparametric Kruskal-Wallis test, followed by multiple comparison evaluation by Dunn's posttest (§ p<0.05 to control cells, *∗*p<0.05 versus 4°C, † p<0.05 versus 19°C, £ p<0.05 versus 27°C, ‡ p<0.05 versus 37°C).

To test the influence of temperature on cell survival after 24 h preservation in hypoxia, we measured LDH release (an indicator of necrotic cell death) from cells preserved in UW at 4°C, 19°C, 27°C, 32°C, or 37°C. Results showed that while basal LDH release level in controls was 17.1 ± 0.8%, it increased significantly at 4°C (37 ± 3%) and at 19°C (40.2 ± 3.3%) groups, indicating increased cell mortality. Conversely, intermediate temperatures significantly decreased cellular necrosis: 27°C (12.2 ± 1%), 32°C (12.9 ± 1%), and 37°C (13.8 ± 1.2 %) (Figure 2(a); n = 8 per group).

### 3.3. Mitochondrial Activity after IR

At the end of 24 h preservation in UW at either 4°C, 19°C, 27°C, 32°C, or 37°C in hypoxia, followed by 24 h reoxygenation at 37°C in completed DMEM, we measured mitochondrial dehydrogenase activity (complex II of the mitochondrial respiratory chain). We observed that while preservation at 4°C and 37°C induced important decrease in complex II activity (45.9 ± 1.1% and 33.7 ± 0.6% of control, respectively), intermediate temperatures induced a significantly higher activity, 19°C (63.8 ± 0.9%) and 27°C (63 ± 0.5%), the highest level of protection being brought about by 32°C (78.5 ± 0.5%; Figure 2(b); n = 16* per* group). Finally, using the same experimental protocol, we showed that while 4°C hypoxic preservation severely decreased ATP levels (33 ± 4% of control cells), (i) 37°C preserved ATP production (81 ± 2%) and 19°C, 27°C and (ii) 32°C yielded higher ATP production, compared to control (respectively, 144 ± 3%, 130 ± 2%, and 206 ± 7% of control cells; Figure 2(c); n = 16* per* group).

### 3.4. Innate Immunity Analysis after In Vitro Blood Reoxygenation at 37°C

**Figure 3:**
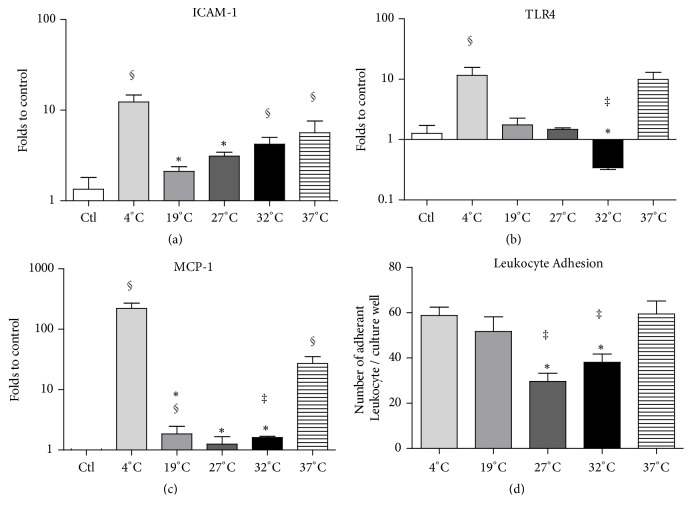
Effects of preservation temperature on endothelial cells phenotype: the influence of preservation at various temperatures was evaluated at the transcriptomic level by RTqPCR, measuring the expression of endothelial activation markers ICAM-1 (a), TLR4 (b), and MCP-1 (c). Results are expressed as fold-changes versus control cells (n = 3 per group). Endothelial activation was also evaluated by measuring the number of leukocytes adherent to the cell layer after 2 h reoxygenation with whole blood (d). Results are expressed in mean ± SEM of number of adherent leukocyte/culture well (n = 3 per group). Significant statistical data (p<0.05) were calculated using nonparametric Kruskal-Wallis test + multiple comparison evaluation by Dunn's posttest (§ p<0.05 to control cells, *∗*p<0.05 versus 4°C, † p<0.05 versus 19°C, £ p<0.05 versus 27°C, ‡ p<0.05 versus 37°C).

To investigate the innate immunity signaling at the different preservation temperatures, cells were preserved for 24 h in UW 0% O_2_ at 4°C, 19°C, 27°C, 32°C, or 37°C, followed by 2 h reoxygenation at 37°C, performed with porcine blood, and then submitted to transcriptional analysis. Endothelial activation marker ICAM-1 was significantly overexpressed in cells preserved at 4°C (12.3 ± 2.4 folds) while at 19°C, 27°C, 32°C, and 37°C preservation the increase was significantly less (2.1 ± 0.3, 3.1 ± 0.3, 4.2 ± 0.8, and 5.6 ± 1.9 folds, respectively; Figure 3(a); n = 3* per* group). Danger signal receptor TLR4 was significantly overexpressed in the 4°C (7.7 ± 4.1 folds) and 37°C (9.9 ± 3.1 folds) groups, while it was unchanged at 19 and 27°C and significantly reduced at 32°C (0.3 ± 0.02 folds) (Figure 3(b); n = 3* per* group). Finally, the innate immune system activator MCP-1 was significantly overexpressed in cells preserved at 4°C (220 ± 49.6 folds) and 37°C (27.1 ± 8 folds), while its expression remained similar to the control level in all other groups (Figure 3(c); n = 3* per* group).

### 3.5. Leukocytes Adhesion after In Vitro Blood Reoxygenation

We further quantified leukocyte adhesion on endothelial cells subjected to 24 h hypoxic preservation in UW at different temperatures, followed by 2 h reoxygenation at 37°C with porcine blood. We found a higher number of adherent leukocytes in 4°C (59 ± 4), 19°C (52 ± 7), and 37°C (59 ± 6) groups, compared to 27°C (30 ± 4) and 32°C (38 ± 4 cells) groups (Figure 3(d); n = 3* per* group).

### 3.6. Cellular Morphological Integrity

**Figure 4:**
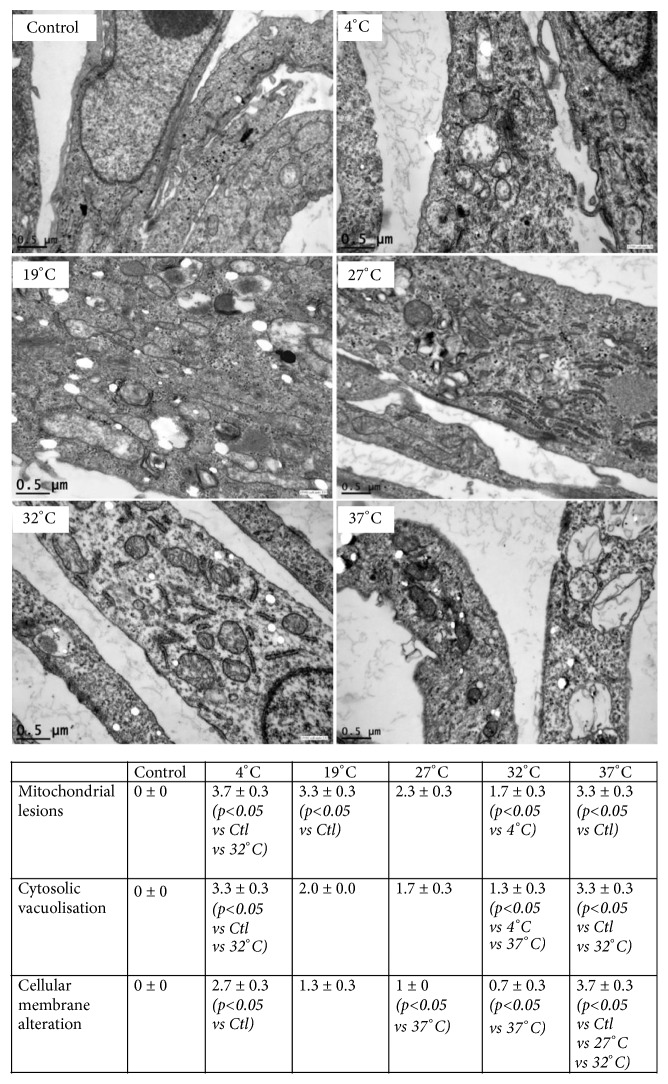
Effects of preservation temperature on endothelial cells morphological integrity. Electronic microscopy analysis of endothelial cells after preservation in UW at various temperatures followed by 24 h of reoxygenation 37°C in culture medium, (n = 3 per group). Results are expressed as mean ± SEM (n = 3 per condition) of percentages of lesions by field: scores: 0 = no alteration, 1 = lesions between 1-25%, 2 = lesions between 26-50%, 3= lesions between 51-75%, and 4 = lesions >76%. Significant statistical data (*∗*p<0.05) were calculated using nonparametric Kruskal-Wallis test + multiple comparison evaluation by Dunn's posttest.

By electron microscopy analysis, performed at the end of 24 h hypoxia preservation in UW (4°C, 19°C, 27°C, 32°C or 37°C) followed by 24 h reoxygenation at 37°C in completed DMEM, we showed that, as compared to normal control cells, cells preserved at 4°C had smaller mitochondria, with fewer visible crests. These lesions were also observed in the 37°C group, exhibiting with small and lysed mitochondria, cytosolic vacuolization, and plasma membrane alteration. However, fewer mitochondria were lysed at 19°C, and 27°C and 32°C temperatures positively impacted mitochondria number and aspect with maintenance of longer mitochondria with visible crests and cytosolic integrity (Figure 4) (n = 3 per group).

### 3.7. Evaluation of Morphology, Leukocyte Infiltration, and Apoptosis after Ex Vivo Kidney Preservation

**Figure 5:**
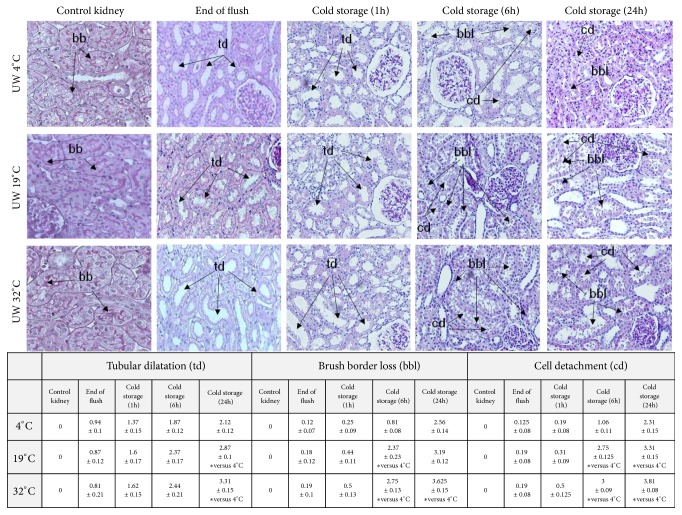
Effect of* ex vivo* kidney preservation at 4°C, 19°C or 32°C on renal histological injuries. Representative pictures of kidney histology for the evaluation of tubular dilatation (dt), brush border loss (bbl), and cell detachment (cd) injuries by histological analysis (PAS coloration) in renal tissue from control time to 1 h, 6 h, and 24 h of organ preservation at 4°C, 19°C, and 32°C (n = 3 per group). Results are expressed as mean ± SEM of percentage of lesions by field; scores: 0 = no alteration, 0.5 = lesions <5%, 1 = lesions 6-10%, 2 = lesions between 11-25%, 3 = lesions between 26-50%, 4 = lesions between 51 and 75%, and 5 = lesions >76%. Statistical significance (*∗*, p<0.05) was calculated using nonparametric Kruskal-Wallis test + multiple comparison correction by Dunn's posttest.

In addition to* in vitro* experiments we performed* ex vivo* determinations by submitting porcine kidneys to UW flush, followed by preservation in UW during 24 hours under static conditions at 4°C, 19°C, and 32°C. Histological analysis showed that, first, tubular dilatation was similar at the end of the UW flush and after 1 h of conservation for all temperature groups, but then gradually increased after 6 h and 24 h of conservation, indicating a degradation of the tissue structure along preservation time. Of note, tubular dilatation areas number was significantly higher in the 19 and 32°C groups versus the 4°C group after 24 h of preservation. Similarly, brush border loss and cell detachment (cellular necrotic injuries) increased significantly from 6 h to 24 h after conservation in the 19°C and 32°C groups while damage remained limited in the 4°C group (Figure 5; n = 3 per group).

**Figure 6:**
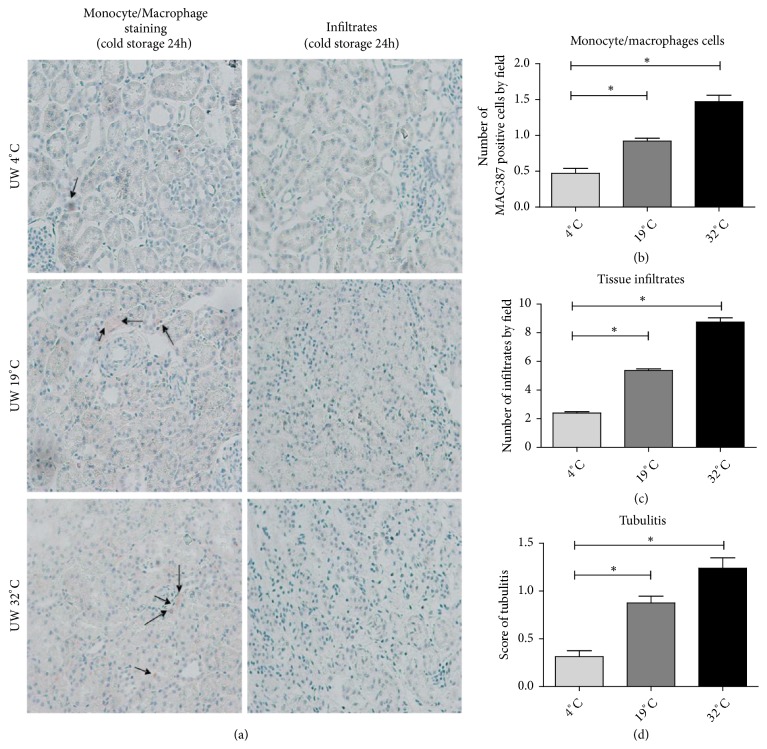
Effect of* ex vivo* kidney preservation at 4°C, 19°C, or 32°C on tissue leukocyte infiltration. (a) Representative pictures of monocyte/macrophage infiltration and leukocyte infiltrates in the tissue after 24 h of storage at 4°C, 19°C, and 32°C (n = 3 per group). (b) Number of monocyte/macrophage cells (MAC387 positive staining) by field. (c) Number of infiltrates by field. (d) Score of tubulitis. Statistical significance (*∗*p<0.05) was calculated using nonparametric Kruskal-Wallis test, corrected by multiple comparison Dunn's posttest.

After 24 h UW preservation at 19°C, the number of infiltrated monocytes/macrophages (MAC387 staining), cellular infiltrates, and the score of cortical tubulitis increased significantly compared to 4°C (p<0.05). These indicators were further increased at 32°C (p<0.05 versus 4°C and versus 19°C) (Figures 6(a) and 6(b)) (n = 3 per group).

**Figure 7:**
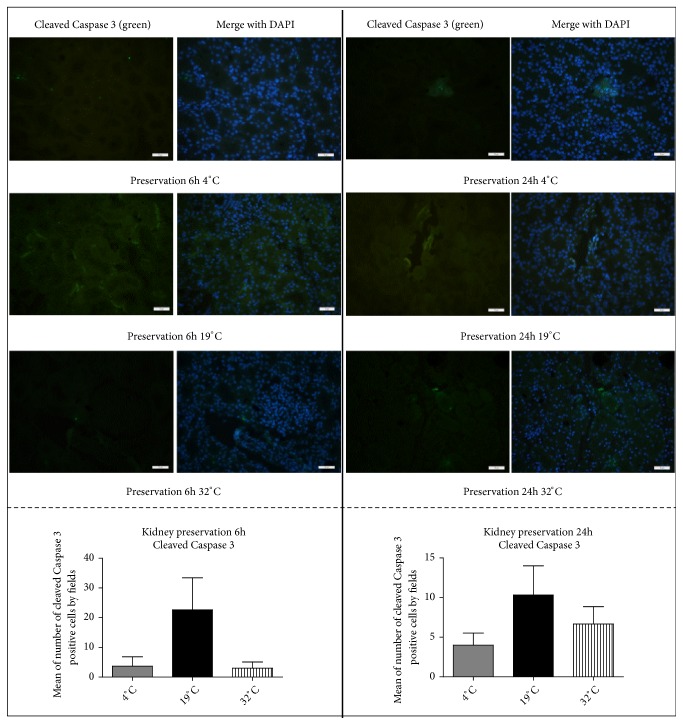
Effect of* ex vivo* kidney preservation at 4°C, 19°C, or 32°C on apoptosis. Left panel: representative pictures and histogram of the number of cleaved caspase 3 positive cells (mean of positive cells per field), as detected by immunohistofluorescence on kidney tissue after 6 h of preservation in UW at 4°C, 19°C and 32°C (n = 3). Right panel: representative pictures and histogram of number of cleaved caspase 3 positive cells (mean of positive cells per field), detected by immunohistofluorescence on kidney tissue after 24 h of preservation in UW at 4°C, 19°C, and 32°C (measure on 10 fields per slide. n = 3 per group).

Furthermore, we observed a trend for a higher number of cleaved caspase 3 positive cells in the kidneys preserved for 6 h and 24 h at 19°C or 32°C compared to 4°C (Figure 7) (n = 3* per* group).

**Figure 8:**
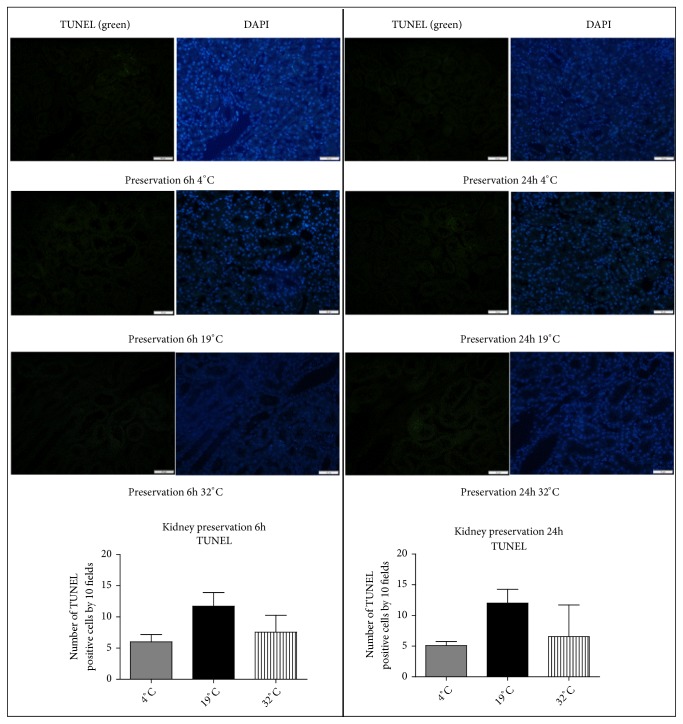
Effect of* ex vivo* kidney preservation at 4°C, 19°C, or 32°C on apoptosis (TUNEL evaluation). Representative pictures and histogram of number of TUNEL positive cells, detected by immunohistofluorescence on kidney tissue after 6 h (left panel) and 24 h (right panel) of preservation in UW at 4°C, 19°C, and 32°C (n = 3). Statistical significance (*∗*p<0.05) was calculated using nonparametric Kruskal-Wallis test and multiple comparison Dunn's posttest.

In addition, we observed a trend for a higher number of apoptotic cells (DNA fragmentation detected by TUNEL staining) in the kidneys preserved during 6 h and 24 h at 19°C compared to 4°C (Figure 8; n = 3* per* group).

## 4. Discussion

In preservation-transplantation, ischemia-reperfusion injury (IRI) is an inevitable event, caused primarily, although not exclusively, by hypothermia and hypoxia during the ischemic phase and by return to normal temperature and reoxygenation during the reperfusion phase. Circumventing the deleterious consequences of hypothermia is one pivotal objective to improve graft outcome. In the current study, we tested different preservation temperatures in an* in vitro* model of IR using endothelial cells, because this is the first cell type to be affected during the IR syndrome, with major consequences on vasculature integrity and function [25–28].

Of note, the temperature exerted a weak influence on O_2_ level decrease in the culture flask atmosphere or in the UW preservation medium.

*In vitro* analyses of cell survival (Figure S1) showed that cell preservation at intermediate temperatures (19°C, 27°C and 32°C) was able to improve cell survival, compared to both extremes, i.e., 4°C/hypothermia (the farthest condition compared to physiological one, despite strongly reducing cell metabolism) and 37°C/normothermia (the condition linked with the higher metabolic demand). In order to characterize the effect of normothermic reoxygenation, we evaluated mitochondrial function and observed that both 4°C and 37°C hypoxic preservations severely impaired complex II activity and ATP production. Mitochondrial complex II oxidizes succinate to fumarate and reduces ubiquinone, thereby creating a direct link between the tricarboxylic acid (TCA) cycle (also known as citric acid or Krebs cycle) and the respiratory chain, the main ATP provider in eukaryotic cells [29]. In this model, we also noted that intermediate temperatures preserved ATP production better than 4°C. Within the range of tested temperatures, 32°C was the most efficient to improve cellular ability to produce ATP. Several studies have confirmed the importance of a relative preserved ATP level for cells exposed to hypoxia and hypothermia, followed by rewarming and reoxygenation, to maintain cell viability and optimal graft function [30, 31]. Indeed, we showed that ATP level is negatively correlated to LDH release, itself associated with level of cell integrity failure [30]. Use of intermediate temperature was also associated with weaker mitochondria and cytosolic alterations, as shown by electron microscopy. Such data suggest that antagonistic processes that predominate under different conditions were able to adapt mitochondrial morphology and dynamics to the bioenergetic requirements of the cell [32].

IRI are associated with vascular dysfunction, increased vascular permeability, endothelial cell inflammation, and macrophage infiltration in the damaged tissue, such features representing the prototypical response of the macro- and microvasculature to a stress or an injury [33]. Thus, preservation of the endothelial wall integrity is crucial to improve function recovery and limit inflammation [9]. Furthermore, expression of immune cell receptors and chemokines by activated endothelial cells induces monocyte and neutrophil adhesion, triggers inflammation and is partially responsible for the “no-reflow” phenomenon through endothelial cell detachment [34, 35]. We sought to further characterize the effects of the different preservation temperatures on ICAM-1, TLR4, and MCP-1 transcriptional levels after endothelial cells reoxygenation with whole blood. ICAM-1 is a membrane receptor which favors leukocyte adhesion before infiltration. In the current study, expression of this molecule was significantly increased after preservation at 4°C, confirming the link between severe hypothermic preservation and leukocyte infiltration. On the other hand, preservation using intermediate temperatures (19°C, 27°C, and 32°C) was able to significantly reduce this expression [36]. The TLR4 exerts a crucial role during IRI. TLR4 is an established danger signal receptor, the increase of which is associated with an important proinflammatory response in IRI [24, 37, 38] and characterized by increased production of cytokines such as MCP-1. The activation of TLR4 leads to an increased production of proinflammatory cytokines and adhesion molecules. In our hands, TLR4 expression was increased at both 4°C and 37°C, while it remained at control levels for intermediate temperatures of 19°C and 27°C and was even decreased below control levels at 32°C. This result suggests that endothelial cells preserved at these temperatures are less exposed to danger signals and/or to activation of the innate immune system. To confirm our results, we measured MCP-1 mRNA expression. MCP-1 is implicated early after IR and contributes mainly to leukocyte attraction [39]. In the current study, increased expression of MCP-1 was detected in the 4°C and 37°C conditions, compared with control and intermediate temperature (19°C, 27°C, and 32°C). Collectively, our results suggest that endothelial cells preserved at temperatures ranging from 19°C to 32°C are less likely to foster the leukocyte adhesion and inflammation and consequently innate immunity activation.

We sought to confirm these observations by the quantification of adherent leukocytes after 2h reoxygenation with blood. In accordance with previous results, 4°C and 37°C preserved cell monolayers displayed an important number of adherent leukocytes. Surprisingly, preservation at 19°C did not reduce leukocyte adhesion, but a lower number of adherent leukocytes was observed at both 27°C and 32°C preserved cells compared to other conditions.

Our* in vitro* study suggests that intermediate preservation temperatures between 19°C and 32°C are more conducive to both the preservation of endothelial cell integrity and to the limitation of innate immunity during IRI. Our results confirm Post and coworkers' work, reporting the effect of different temperatures and preservation solution on endothelial cells viability and function [40]. In this study, authors showed that UW was more protective at 20°C than at lower temperatures. However, the authors were not able to show superiority of temperatures above 20°C, except in cell culture medium. Furthermore, Post and coworkers compared preservation temperatures (4°C to 37°C) and observed no influence of low-potassium (extracellular-like medium) or high potassium (intracellular-like medium) preservation solution in terms of cell injuries [40]. However, careful comparison of the study reveals conceptual differences. While our study used primary endothelial cells extracted from the microvasculature of the kidney and measured the effects of both preservation and reperfusion, study from Post and his group used venous endothelial cells, considered less sensitive to hypoxia [40]. In addition, the authors incubated the cells in a hyperoxygenated environment (95% O_2_, 5% CO_2_), far from hypoxic the conditions generally prevailing during organ preservation.

Of note, we used the UW preservation solution displaying a high concentration of potassium and designed to be used at 4°C. Importantly, high concentrations of potassium are known to be toxic for cells. Lee et al. showed in a piglet* ex vivo *model that lower potassium concentration (25 versus 125 mEq/L) is superior for myocardial endothelial cell function at 37°C [41]. Similarly, in a human bypass saphenous vein model, De Caterina et al. showed that high potassium concentration in cardioplegic solutions inhibit prostacyclin production as a marker of endothelial function, without any observed endothelial detachment [42]. Indeed, potassium has been described to impact endothelial cell-derived hyperpolarizing factor mediated vasodilatation at 37°C [43, 44]. Similarly, we showed in our study that high potassium concentration is deleterious* in vitro* at 4 and 37°C (versus 19, 27, and 32°C) for cell integrity, which is in accordance with the literature. However,* ex vivo*, this hyperpotassic effect seems not worse at 4°C than at other tested temperatures (19 and 32°C) in terms of tissue integrity. This variation may be due to the fact that a part of the effects triggered by high potassium (such as vasodilatation) are dependent on the endothelial cell environment and interactions with other cell types within the tissue.

Nonetheless, both studies bring compelling arguments for the use of mild temperature to preserve organs. Our cell model was fruitful to mimic a particular IRI situation. However,* in vitro* results cannot be easily extrapolated to the patient level.

Thus, we moved forward with a preclinical pig model of kidney preservation for the second part of this study. This model is very close to the clinical situation and pig kidneys share strong anatomical and physiological similarities with human ones [26, 28]. In addition, the kidney is a multifunctional organ, not only eliminating metabolic waste, but also regulating the internal milieu* via* hydroelectrolytic balance. During its development toward adult kidney, different progenitor cells differentiate into more than 26 different cell types, exhibiting a variety of functions, metabolism levels and tolerance to IRI. Histological analysis of the first time points (immediate postflush or 1h preservation) suggested no effect of the preservation temperature. Moreover, increasing the preservation time to 6 and 24 hours did not confirm the apparent superiority of intermediate temperatures. This observation is associated with increased necrosis (cell detachment), tubulitis, and monocytes/macrophages infiltration, in tissues preserved 6h and 24h at 19°C and 32°C versus 4°C storage. Nevertheless, we observed no statistical differences on apoptotic cells detection (cleaved caspase-3 positive cells and TUNEL staining) in the tissues preserved at 4°C, 19°C, or 32°C (despite an increase trend towards in the 19°C group).

This discrepancy between* in vitro* and* ex vivo* data highlights that (i) the choice of the experimental model is of critical importance in order to generate data relevant for translational research, (ii)* in vitro* data need to be thoroughly checked in complementary* ex vivo* or* in vivo* models, (iii) although moderate hypothermia yields interesting results regarding organ preservation, it will require the concomitant development of new solutions, specifically designed for maintaining the viability of an organ at the chosen temperatures. In addition, technological limitations will have to be surmounted. Overall, in order to enforce the static-to-dynamic/hypo-to-(sub)normothermic preservation paradigm shift, transplant community faces scientific, methodologic, and economic challenges.

A great deal of research is still needed to provide more rigorous information on pathophysiological mechanisms [12, 25]. Promoting recovery by introducing an intervention during perfusion is a fascinating area of research. In addition, endothelium is likely a research area of interest.

In this vein, a pilot study demonstrated the superiority of Lifor Preservation Medium (an organ preservation medium containing sugars, amino acids, salts, buffers, colloids, fatty acids, antioxidants, vitamins, dextran, and an oxygen carrier) at room temperature perfusion compared to Belzer machine perfusion at both room temperature and 4°C, in a porcine model of uncontrolled donation after circulatory death [45]. It was also shown in a rat model that increasing the liver perfusion temperature to 21°C with HTK solution allowed an hepatic functional capacity recovery, which was more efficient than perfusion at 4°C or 12°C [17].

Several reports support the notion that the preservation setting at other temperatures that 4°C is still not feasible during the whole ischemic period [11, 12, 46]. Alternatives using combination of short-term abdominal circulation, short* ex vivo* normothermic perfusion, and/or progressive organ rewarming, after cold storage may have to be considered [46]. For instance, in the rat liver, a short-term resuscitation with oxygenated machine perfusion (HTK, 12°C, 2 hours), with subsequent cold storage for 16 hours at 4°C, was shown to induce a better tissue ATP recovery compared to 4°C and 22°C [47]. Subnormothermic machine perfusion for preservation of porcine kidneys at 21°C [48] or human livers at 20°C or 30°C seems to be a promising preservation method with the potential to improve functional parameters [49, 50]. Moreover, controlled oxygenated rewarming (COR) organ after cold storage is another alternative, with a prevention of mitochondrial initiation of cellular apoptosis, as evidenced by a reduced activation of caspase 9 [23]. Gradual rewarming at 10°C and 25°C during machine perfusion after static cold preservation reduced rat kidney IRI [51]. Subnormothermia or normothermia preservation condition could be an interesting strategy to prepare or help to condition the graft more physiologically. However this should be used with adequate technology and adapted conditions: (1) perfusion solution (e.g., whole blood versus leucocyte-free blood versus reconstituted washed red blood cells); (2) temperature (room temperature to normothermia); (3) judicious choice of time during the preservation phase (e.g., at the beginning of preservation process or few hours before transplantation).

One limit of our study was the use of the “intracellular-type” UW solution with high potassium content, designed and used for low temperature preservation, bringing difficulties to discriminate temperature and potassium effects in our models. However, at least under static condition, there is no preservation solution designed for temperature above 10°C. Thus, in order to investigate the effect of temperature* per se*, we chose to alter only one parameter of graft storage, leaving solution and oxygenation unchanged. This can limit the impact of our observations, while allowing for a clearer definition of temperature impact, in addition to the reduction in number of experimental groups and variables. Another limit is that we did not perform a kinetic investigation during storage* in vitro. *However, our foremost objective was to discriminate between the temperatures, so we did select an extended cold ischemia time (24 h), for which we knew [52, 53] that injuries would be severe enough to observe differences between the conditions. Nonetheless, this work allowed us to select the most pertinent temperatures to be investigated* ex vivo*, at different storage times. However, while cold storage is still widely used in clinics, alternative strategies to optimize organ quality during its preservation need to be developed. Furthermore, the suitable temperature does depend on the preservation solution composition; our study is facing the complexity of the organ preservation issue where all the parameters (temperature, preservation solution composition, oxygen, static or perfusion preservation, etc.) are dependent factors which have combined impacts on the tissue integrity and need to be optimized to fully answer to the targeted tissue metabolic demand.

## 5. Conclusion

The demography of organ donors is changing, pushing the transplant community to move towards new graft preservation paradigms. Based on our work and others, the versatile use of mild hypothermia, subnormothermia, or normothermia is a technological barrier that the transplant community has to overcome. Such conditions require sophisticated technologies and adapted preservation solutions since available technologies do not meet the relevant criteria. Our work also advocates that extrapolation of* in vitro* observations to* ex vivo* or* in vivo* settings must be carefully evaluated.

Critical in normothermia, oxygen is progressively understood as beneficial, if not required, even at the lowest preservation temperatures. On the other hand, normothermic preservation emerges as the next-to-come standard for higher-risk organs, while hypothermic preservation still prevails for standard and low-risk organs. From this viewpoint, the preservation temperature will have to be adapted to the organ and the donor, as well as to the perfusion regimen and conditions, including oxygen level, preservation solution, and its energetic metabolites. Research is being directed toward optimized graft-tailored preservation and repair is crucial and as important as management of acute rejection in the early days of transplantation. It deserves to be tackled with high priority and the same urgency.

## Data Availability

All the data supporting our finding are included in the main manuscript and in the supplementary data.

## Supplementary Materials

Supplementary Materials Figure S1: percentage of* in vitro* injuries induced by various tested preservation temperatures on endothelial cells. Table S1: primers used for real-time RT-PCR in porcine blood leukocytes and renal cortex tissue.
