# Supplementary material for: Influence of Hypoxic Preservation Temperature on Endothelial Cells and Kidney Integrity
Source: Biomed Res Int. 2019 Jun 4;2019:8572138. doi: 10.1155/2019/8572138 (PMC6582897; doi:10.1155/2019/8572138)
Supplement: Supplementary Materials — Figure S1: percentage of in vitro injuries induced by various tested preservation temperatures on endothelial cells. Table S1: primers used for real-time RT-PCR in porcine blood leukocytes and renal cortex tissue. [file 8572138.f1.pdf]

Supplementary Materials

Figure S1: Percentage of *in vitro* injuries induced by various tested preservation temperatures on endothelial cells

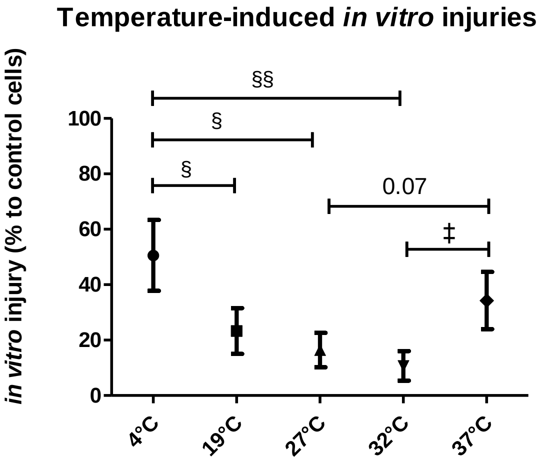

Figure S1: Percentage *of in vitro injuries induced by various tested preservation temperatures on endothelial cells. Scores were obtained by combination of in vitro evaluation results (LDH release, mitochondrial complex II activity, ATP measurement, ICAM-1 mRNA, MCP-1 mRNA, TLR4 mRNA and Leukocyte adhesion quantifications). Results are expressed % versus control cells (mean ± SEM). Significant statistical data (p<0.05) were calculated using nonparametric Kruskal Wallis test + multiple comparison evaluation by Dunn's post test (§ p<0.05 to Control cells, *p<0.05 versus 4°C, † p<0.05 versus 19°C, £ p<0.05 versus 27°C, ‡ p<0.05 versus 37°C).*

Table S1: Primers used for real-time RT-PCR in porcine blood leukocytes and renal cortex tissue.

**Target**	**Primer Sequence**
L19 (RPL19)	Forward primer : 5'-AATCGCCAACGCCAACTC-3'
	Reverse primer : 5'-CAGCCCATCTTTGATCAGCTT-3'
ICAM-1	Forward primer : 5'-GGCTGTGCACTGCAACAAGA-3'
	Reverse primer : 5'-TGTGGCAATGCCAAATCCT-3'
MCP-1	Forward primer : 5'-TCTCCAGTCACCTGCTGCTAT-3'
	Reverse primer : 5'-TGCTTCTTTAGGACACTTGCTG-3'
TLR4	Forward primer : 5'-GCTTTTTGTGGGCTGCAAA-3'
	Reverse primer : 5'-GGAGTAGATAACAAAGGCGTCATAGG-3'

Abbreviations: L19 ribosomal protein gene (RPL19), Intercellular Adhesion Molecule-1 (ICAM-1), monocyte chemoattractant protein 1 (MCP-1 also call CCL2), Toll Like Receptor-4 (TLR4)
